# (1*S*,3*R*,8*S*,9*R*,10*S*)-2,2-Dichloro-3,7,7,10-tetra­methyl-9,10-ep­oxy­tricyclo­[6.4.0.0^1,3^]dodeca­ne

**DOI:** 10.1107/S1600536810045344

**Published:** 2010-11-10

**Authors:** Ahmed Benharref, Lahcen El Ammari, Daniel Avignant, Abdelghani Oudahmane, Moha Berraho

**Affiliations:** a Laboratoire de Chimie Biomoléculaires, Substances Naturelles et Réactivité, URAC16, Faculté des Sciences, Semlalia, BP 2390 Bd My Abdellah, 40000 Marrakech, Morocco; b Laboratoire de Chimie du Solide Appliquée, Faculté des Sciences, Avenue Ibn Battouta, BP 1014 Rabat, Morocco; c Université Blaise Pascal, Laboratoire des Matériaux Inorganiques, UMR CNRS 6002, 24 Avenue des Landais, 63177 Aubière, France

## Abstract

The title compound, C_16_H_24_Cl_2_O, was synthesized from β-himachalene (3,5,5,9-tetra­methyl-2,4a,5,6,7,8-hexa­hydro-1*H*-benzocyclo­heptene), which was isolated from the essential oil of the Atlas cedar (*cedrus atlantica*). The mol­ecule forms an extended sheet of two fused rings which exhibit different conformations. The six-membered ring has a half-chair conformation, while the seven-membered ring displays a chair conformation; the dihedral angle between the two rings is 38.2 (1)°.

## Related literature

For the isolation of β-himachalene, see: Joseph & Dev (1968 ▶); Plattier & Teiseire (1974 ▶). For the reactivity of this sesquiterpene, see: Lassaba *et al.* (1998 ▶); Chekroun *et al.* (2000 ▶); El Jamili *et al.* (2002 ▶); Sbai *et al.* (2002 ▶); Dakir *et al.* (2004 ▶). For its biological activity, see: Daoubi *et al.* (2004 ▶). For ring puckering parameters, see: Cremer & Pople (1975 ▶).
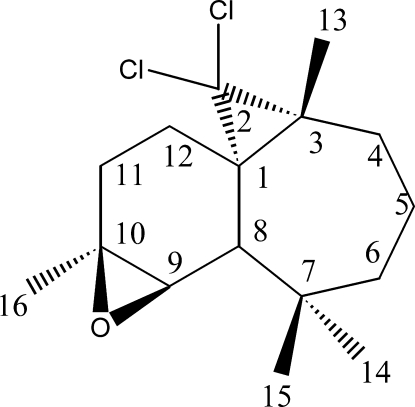


## Experimental

### 

#### Crystal data

C_16_H_24_Cl_2_O
*M* *_r_* = 303.24
Orthorhombic, 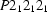

*a* = 8.4995 (3) Å
*b* = 10.2461 (4) Å
*c* = 18.1656 (6) Å
*V* = 1581.98 (10) Å^3^
*Z* = 4
Mo *K*α radiation
μ = 0.40 mm^−1^
*T* = 298 K
0.67 × 0.41 × 0.26 mm

#### Data collection

Bruker APEXII CCD diffractometer
Absorption correction: multi-scan (*SADABS*; Bruker, 2008) ▶ *T*_min_ = 0.609, *T*_max_ = 0.745
7171 measured reflections
3369 independent reflections
2830 reflections with *I* > 2σ(*I*)
*R*_int_ = 0.025

#### Refinement

*R*[*F* ^2^ > 2σ(*F* ^2^)] = 0.040
*wR*(*F* ^2^) = 0.106
*S* = 1.01
3369 reflections
177 parameters
H-atom parameters constrained
Δρ_max_ = 0.22 e Å^−3^
Δρ_min_ = −0.29 e Å^−3^
Absolute structure: Flack & Bernardinelli (2000 ▶), 1423 Friedel pairs
Flack parameter: 0.04 (7)

### 

Data collection: *APEX2* (Bruker, 2009 ▶); cell refinement: *SAINT* (Bruker, 2009 ▶); data reduction: *SAINT*; program(s) used to solve structure: *SHELXS97* (Sheldrick, 2008 ▶); program(s) used to refine structure: *SHELXL97* (Sheldrick, 2008 ▶); molecular graphics: *ORTEP-3 for Windows* (Farrugia, 1997 ▶); software used to prepare material for publication: *WinGX* (Farrugia, 1999 ▶).

## Supplementary Material

Crystal structure: contains datablocks I, global. DOI: 10.1107/S1600536810045344/im2241sup1.cif

Structure factors: contains datablocks I. DOI: 10.1107/S1600536810045344/im2241Isup2.hkl

Additional supplementary materials:  crystallographic information; 3D view; checkCIF report

## Figures and Tables

**Fig. 1.**
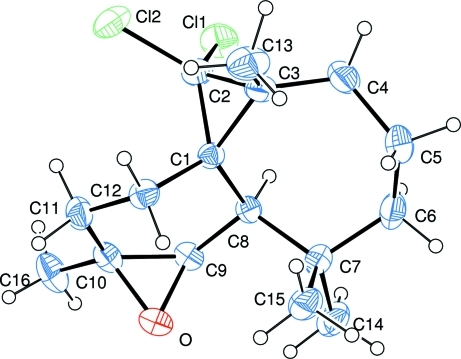
Molecular structure of the title compound with the atom-labelling scheme. Displacement ellipsoids are drawn at the 30% probability level. H atoms are represented as small spheres of arbitrary radii.

## supplementary crystallographic information

### Comment

The bicyclic sesquiterpene β-himachalene is the main constituent of the essential oil of the Atlas cedar (*Cedrus atlantica*) (Joseph & Dev (1968); Plattier & Teiseire(1974)). The reactivity of this sesquiterpene and its derivatives has been studied extensively by our team in order to prepare new products having biological proprieties.(Lassaba *et al.*, 1998; Chekroun *et al.*, 2000; El Jamili *et al.*, 2002; Sbai *et al.*, 2002; Dakir *et al.*, 2004). Indeed, these compounds were tested, using the food poisoning technique, for their potential antifungal activity against phytopathogen *Botrytis cinerea* (Daoubi *et al.*, 2004). Thus the action of one equivalent of dichlorocarbene, generated *in situ* from chloroform in the presence of sodium hydroxide as base and n-benzyltriethylammonium chloride as catalyst, on β-himachalene produces only (1*S*,3*R*,8*S*)-2,2-dichloro-3,7,7,10- tetramethyltricyclo[6,4,0,0^1,3^]dodec-9-ene (*X*) (El Jamili *et al.*, 2002). Treatement of (*X*) with one equivalent of *meta*-chloroperbenzoic acid (mCPBA) leads to a mixture of two diastereisomers: (1*S*, 3*R*, 8S, 9S, 10*R*)-2,2-dichloro-9–10-epoxy-3,7,7,10-tetramethyl- tricyclo[6.4.0.0^1,3^]dodecane (*Y*) and its isomer (1*S*, 3*R*, 8S, 9*R*, 10S)-2,2-dichloro-9–10-epoxy-3,7,7,10-tetramethyl-tricyclo[6.4.0.0^1,3^]dodecane (*Z*) in an over-all yield of 80% and 30:70 ratio. In a previous work (Sbai *et al.*, 2002), we have determined the structure and the stereochemistry of *Y.* In this paper we present the absolute configuration of *Z* established by single-crystal X-ray diffraction analysis. The molecule is built up from two fused six-membered and seven-membered rings (Fig. 1). The six-membered ring has a half chair conformation, as indicated by the total puckering amplitude QT = 0.513 (2) Å and spherical polar angle θ = 125.9 (2)° with φ = 138.1 (4)°, whereas the seven-membered ring displays an aproximate chair conformation with QT = 0.783 (3) Å, θ = 31.9 (3)°, φ2 = -50.3 (4)° and φ3 =-78.3 (2)° (Cremer & Pople, 1975). Owing to the presence of Cl atoms, the absolute configuration could be fully confirmed, by refining the Flack parameter (Flack & Bernardinelli (2000)) as C1(*S*), C3(*R*), C8(S), C9(*R*) and C10(*S*).

### Experimental

For the synthesis of compounds (1*S*, 3*R*, 8S, 9S, 10*R*)-2,2-dichloro-9–10- epoxy-3,7,7,10-tetramethyl-tricyclo[6.4.0.0^1,3^]dodecane (*Y*) and its isomer (1*S*, 3*R*, 8S, 9*R*, 10S)-2,2-dichloro-9–10-epoxy-3,7,7,10- tetramethyl-tricyclo[6.4.0.0^1,3^]dodecane (*Z*), a stoichiometric quantity of *m*-chloroperbenzoic acid (*m*-CPBA) was added to a 100 ml flask containing a solution of (1*S*,3*R*,8*S*)-2,2-dicchloro-3,7,7,10- tetramethyltricyclo[6,4,0,0^1,3^]dodec-9-ene (*X*) (500 mg, 1.74 mmol) in CH_2_Cl_2_ (30 ml). The reaction mixture was stirred at ambient temperature for 2 h, then treated with a 10% solution of sodium hydrogencarbonate. The aqueous phase was extracted with ether and the organic phases were dried and concentrated. Chromatography of the residue on silica (hexane/ethyl acetate 97/3) allowed the isolation of both isomers *Y* and *Z* in a pure state. Crystallization of *Z* was carried out at room temperature from a hexane solution.

### Refinement

All H atoms were fixed geometrically and treated as riding with C—H = 0.96 Å (methyl), 0.97 Å (methylene), 0.98Å (methine) with *U*_iso_(H) = 1.2U_eq _(methylene, methine) or *U*_iso_(H) = 1.5*U*_eq_ (methyl).

### Crystal data

**Table:**

C_16_H_24_Cl_2_O	*F*(000) = 648
*M**_r_* = 303.24	*D*_x_ = 1.273 Mg m^−^^3^
Orthorhombic, *P*2_1_2_1_2_1_	Mo *K*α radiation, λ = 0.71073 Å
Hall symbol: P 2ac 2ab	Cell parameters from 3372 reflections
*a* = 8.4995 (3) Å	θ = 2.3–26.9°
*b* = 10.2461 (4) Å	µ = 0.40 mm^−^^1^
*c* = 18.1656 (6) Å	*T* = 298 K
*V* = 1581.98 (10) Å^3^	Prism, colourless
*Z* = 4	0.67 × 0.41 × 0.26 mm

### Data collection

**Table:**

Bruker APEXII CCD diffractometer	3369 independent reflections
Radiation source: fine-focus sealed tube	2830 reflections with *I* > 2σ(*I*)
graphite	*R*_int_ = 0.025
Detector resolution: 8.3333 pixels mm^-1^	θ_max_ = 26.9°, θ_min_ = 2.3°
ω and φ scans	*h* = −9→10
Absorption correction: multi-scan (*SADABS*; Bruker, 2008)	*k* = −13→10
*T*_min_ = 0.609, *T*_max_ = 0.745	*l* = −16→22
7171 measured reflections	

### Refinement

**Table:**

Refinement on *F*^2^	Hydrogen site location: inferred from neighbouring sites
Least-squares matrix: full	H-atom parameters constrained
*R*[*F*^2^ > 2σ(*F*^2^)] = 0.040	*w* = 1/[σ^2^(*F*_o_^2^) + (0.0568*P*)^2^ + 0.1767*P*] where *P* = (*F*_o_^2^ + 2*F*_c_^2^)/3
*wR*(*F*^2^) = 0.106	(Δ/σ)_max_ < 0.001
*S* = 1.01	Δρ_max_ = 0.22 e Å^−^^3^
3369 reflections	Δρ_min_ = −0.29 e Å^−^^3^
177 parameters	Extinction correction: *SHELXL97* (Sheldrick, 2008), Fc^*^=kFc[1+0.001xFc^2^λ^3^/sin(2θ)]^-1/4^
0 restraints	Extinction coefficient: 0.073 (4)
Primary atom site location: structure-invariant direct methods	Absolute structure: Flack & Bernardinelli (2000), 1423 Friedel pairs
Secondary atom site location: difference Fourier map	Flack parameter: 0.04 (7)

### Special details

**Table:**

Geometry. All e.s.d.'s (except the e.s.d. in the dihedral angle between two l.s. planes) are estimated using the full covariance matrix. The cell e.s.d.'s are taken into account individually in the estimation of e.s.d.'s in distances, angles and torsion angles; correlations between e.s.d.'s in cell parameters are only used when they are defined by crystal symmetry. An approximate (isotropic) treatment of cell e.s.d.'s is used for estimating e.s.d.'s involving l.s. planes.

### Fractional atomic coordinates and isotropic or equivalent isotropic displacement parameters (Å^2^)

**Table:**

	*x*	*y*	*z*	*U*_iso_*/*U*_eq_	
C1	0.6413 (2)	0.5366 (2)	0.38517 (10)	0.0356 (4)	
C2	0.5713 (3)	0.5121 (2)	0.46043 (13)	0.0457 (5)	
C3	0.6259 (3)	0.3964 (2)	0.41628 (12)	0.0460 (5)	
C4	0.7701 (3)	0.3249 (3)	0.44259 (15)	0.0630 (7)	
H4A	0.7375	0.2445	0.4663	0.076*	
H4B	0.8230	0.3780	0.4792	0.076*	
C5	0.8864 (3)	0.2922 (3)	0.38160 (18)	0.0675 (8)	
H5A	0.9496	0.2180	0.3967	0.081*	
H5B	0.8285	0.2671	0.3378	0.081*	
C6	0.9941 (3)	0.4052 (3)	0.36304 (16)	0.0622 (7)	
H6A	1.0759	0.3720	0.3308	0.075*	
H6B	1.0451	0.4322	0.4083	0.075*	
C7	0.9253 (3)	0.5290 (2)	0.32646 (13)	0.0464 (5)	
C8	0.8050 (2)	0.59920 (19)	0.37903 (11)	0.0359 (4)	
H8	0.8509	0.5936	0.4284	0.043*	
C9	0.7899 (3)	0.7442 (2)	0.36270 (12)	0.0417 (5)	
H9	0.8687	0.7990	0.3871	0.050*	
C10	0.6401 (3)	0.8106 (2)	0.34700 (12)	0.0472 (5)	
C11	0.4936 (3)	0.7314 (2)	0.34176 (14)	0.0513 (6)	
H11A	0.4293	0.7650	0.3019	0.062*	
H11B	0.4344	0.7408	0.3871	0.062*	
C12	0.5260 (3)	0.5877 (2)	0.32837 (12)	0.0438 (5)	
H12A	0.4284	0.5389	0.3314	0.053*	
H12B	0.5692	0.5760	0.2794	0.053*	
C13	0.5035 (4)	0.3054 (3)	0.38394 (17)	0.0677 (8)	
H13A	0.4747	0.2411	0.4199	0.102*	
H13B	0.5462	0.2627	0.3413	0.102*	
H13C	0.4121	0.3547	0.3701	0.102*	
C14	1.0652 (3)	0.6208 (3)	0.31365 (18)	0.0665 (8)	
H14A	1.1444	0.5764	0.2855	0.100*	
H14B	1.1083	0.6469	0.3602	0.100*	
H14C	1.0303	0.6966	0.2872	0.100*	
C15	0.8565 (3)	0.4933 (3)	0.25117 (13)	0.0575 (6)	
H15A	0.8199	0.5710	0.2271	0.086*	
H15B	0.7702	0.4340	0.2577	0.086*	
H15C	0.9362	0.4527	0.2216	0.086*	
C16	0.6208 (4)	0.9531 (3)	0.36539 (17)	0.0711 (8)	
H16A	0.7209	0.9961	0.3619	0.107*	
H16B	0.5809	0.9617	0.4146	0.107*	
H16C	0.5484	0.9924	0.3314	0.107*	
O	0.7511 (2)	0.78441 (15)	0.28799 (9)	0.0529 (4)	
Cl1	0.66972 (9)	0.56107 (8)	0.54165 (3)	0.0691 (2)	
Cl2	0.36713 (8)	0.53272 (8)	0.47590 (4)	0.0698 (2)	

### Atomic displacement parameters (Å^2^)

**Table:**

	*U*^11^	*U*^22^	*U*^33^	*U*^12^	*U*^13^	*U*^23^
C1	0.0372 (10)	0.0357 (10)	0.0339 (9)	0.0002 (9)	0.0019 (9)	−0.0013 (8)
C2	0.0485 (11)	0.0517 (13)	0.0369 (11)	−0.0012 (10)	0.0050 (10)	0.0004 (10)
C3	0.0565 (13)	0.0393 (12)	0.0423 (11)	−0.0039 (10)	0.0074 (11)	0.0030 (9)
C4	0.0839 (18)	0.0461 (14)	0.0591 (16)	0.0145 (13)	0.0087 (14)	0.0109 (12)
C5	0.0754 (18)	0.0448 (14)	0.0822 (19)	0.0203 (13)	0.0098 (16)	0.0030 (13)
C6	0.0529 (15)	0.0563 (16)	0.0774 (17)	0.0187 (12)	0.0092 (14)	−0.0024 (13)
C7	0.0389 (11)	0.0482 (13)	0.0521 (12)	0.0031 (10)	0.0054 (10)	−0.0031 (11)
C8	0.0356 (10)	0.0379 (11)	0.0342 (10)	−0.0006 (9)	−0.0059 (9)	−0.0004 (8)
C9	0.0470 (12)	0.0384 (11)	0.0396 (11)	−0.0050 (9)	−0.0071 (10)	−0.0009 (9)
C10	0.0595 (14)	0.0389 (12)	0.0432 (11)	0.0053 (10)	−0.0069 (11)	0.0019 (9)
C11	0.0449 (13)	0.0572 (15)	0.0517 (14)	0.0121 (11)	−0.0082 (11)	0.0051 (11)
C12	0.0353 (11)	0.0537 (13)	0.0424 (11)	−0.0042 (10)	−0.0034 (9)	−0.0007 (10)
C13	0.081 (2)	0.0477 (14)	0.0743 (18)	−0.0225 (13)	0.0173 (16)	−0.0033 (13)
C14	0.0397 (13)	0.0759 (19)	0.084 (2)	−0.0028 (12)	0.0119 (14)	0.0010 (15)
C15	0.0643 (15)	0.0617 (15)	0.0467 (13)	0.0046 (13)	0.0106 (12)	−0.0106 (11)
C16	0.095 (2)	0.0450 (14)	0.0737 (17)	0.0165 (14)	−0.0216 (17)	0.0008 (13)
O	0.0632 (11)	0.0497 (10)	0.0458 (9)	−0.0048 (8)	−0.0006 (8)	0.0107 (8)
Cl1	0.0888 (5)	0.0842 (5)	0.0342 (3)	0.0068 (4)	−0.0030 (3)	−0.0063 (3)
Cl2	0.0547 (4)	0.0862 (5)	0.0683 (4)	−0.0017 (3)	0.0248 (3)	0.0005 (4)

### Geometric parameters (Å, °)

**Table:**

C1—C2	1.512 (3)	C9—O	1.456 (3)
C1—C12	1.517 (3)	C9—C10	1.472 (3)
C1—C8	1.536 (3)	C9—H9	0.9800
C1—C3	1.550 (3)	C10—O	1.453 (3)
C2—C3	1.505 (3)	C10—C11	1.489 (3)
C2—Cl1	1.769 (2)	C10—C16	1.507 (3)
C2—Cl2	1.770 (2)	C11—C12	1.517 (3)
C3—C4	1.506 (3)	C11—H11A	0.9700
C3—C13	1.516 (4)	C11—H11B	0.9700
C4—C5	1.522 (4)	C12—H12A	0.9700
C4—H4A	0.9700	C12—H12B	0.9700
C4—H4B	0.9700	C13—H13A	0.9600
C5—C6	1.515 (4)	C13—H13B	0.9600
C5—H5A	0.9700	C13—H13C	0.9600
C5—H5B	0.9700	C14—H14A	0.9600
C6—C7	1.546 (3)	C14—H14B	0.9600
C6—H6A	0.9700	C14—H14C	0.9600
C6—H6B	0.9700	C15—H15A	0.9600
C7—C15	1.532 (3)	C15—H15B	0.9600
C7—C14	1.534 (4)	C15—H15C	0.9600
C7—C8	1.574 (3)	C16—H16A	0.9600
C8—C9	1.521 (3)	C16—H16B	0.9600
C8—H8	0.9800	C16—H16C	0.9600
			
C2—C1—C12	114.70 (17)	O—C9—C8	118.51 (17)
C2—C1—C8	119.45 (17)	C10—C9—C8	124.23 (19)
C12—C1—C8	113.09 (17)	O—C9—H9	114.5
C2—C1—C3	58.86 (14)	C10—C9—H9	114.5
C12—C1—C3	120.89 (19)	C8—C9—H9	114.5
C8—C1—C3	119.35 (18)	O—C10—C9	59.71 (14)
C3—C2—C1	61.81 (14)	O—C10—C11	113.26 (19)
C3—C2—Cl1	121.47 (17)	C9—C10—C11	118.93 (18)
C1—C2—Cl1	121.38 (16)	O—C10—C16	114.4 (2)
C3—C2—Cl2	118.75 (17)	C9—C10—C16	120.0 (2)
C1—C2—Cl2	120.64 (16)	C11—C10—C16	116.8 (2)
Cl1—C2—Cl2	107.30 (12)	C10—C11—C12	112.81 (18)
C2—C3—C4	117.7 (2)	C10—C11—H11A	109.0
C2—C3—C13	118.7 (2)	C12—C11—H11A	109.0
C4—C3—C13	112.5 (2)	C10—C11—H11B	109.0
C2—C3—C1	59.33 (14)	C12—C11—H11B	109.0
C4—C3—C1	119.9 (2)	H11A—C11—H11B	107.8
C13—C3—C1	119.2 (2)	C1—C12—C11	110.06 (18)
C3—C4—C5	113.9 (2)	C1—C12—H12A	109.6
C3—C4—H4A	108.8	C11—C12—H12A	109.6
C5—C4—H4A	108.8	C1—C12—H12B	109.6
C3—C4—H4B	108.8	C11—C12—H12B	109.6
C5—C4—H4B	108.8	H12A—C12—H12B	108.2
H4A—C4—H4B	107.7	C3—C13—H13A	109.5
C6—C5—C4	112.7 (2)	C3—C13—H13B	109.5
C6—C5—H5A	109.0	H13A—C13—H13B	109.5
C4—C5—H5A	109.0	C3—C13—H13C	109.5
C6—C5—H5B	109.0	H13A—C13—H13C	109.5
C4—C5—H5B	109.0	H13B—C13—H13C	109.5
H5A—C5—H5B	107.8	C7—C14—H14A	109.5
C5—C6—C7	119.6 (2)	C7—C14—H14B	109.5
C5—C6—H6A	107.4	H14A—C14—H14B	109.5
C7—C6—H6A	107.4	C7—C14—H14C	109.5
C5—C6—H6B	107.4	H14A—C14—H14C	109.5
C7—C6—H6B	107.4	H14B—C14—H14C	109.5
H6A—C6—H6B	107.0	C7—C15—H15A	109.5
C15—C7—C14	107.9 (2)	C7—C15—H15B	109.5
C15—C7—C6	109.4 (2)	H15A—C15—H15B	109.5
C14—C7—C6	106.0 (2)	C7—C15—H15C	109.5
C15—C7—C8	113.74 (18)	H15A—C15—H15C	109.5
C14—C7—C8	108.4 (2)	H15B—C15—H15C	109.5
C6—C7—C8	111.10 (19)	C10—C16—H16A	109.5
C9—C8—C1	110.22 (17)	C10—C16—H16B	109.5
C9—C8—C7	112.54 (18)	H16A—C16—H16B	109.5
C1—C8—C7	116.23 (17)	C10—C16—H16C	109.5
C9—C8—H8	105.6	H16A—C16—H16C	109.5
C1—C8—H8	105.6	H16B—C16—H16C	109.5
C7—C8—H8	105.6	C10—O—C9	60.78 (14)
O—C9—C10	59.51 (14)		
